# Polymeric and Lipid Membranes—From Spheres to Flat Membranes and vice versa

**DOI:** 10.3390/membranes7030044

**Published:** 2017-08-15

**Authors:** Mariia S. Saveleva, Ekaterina V. Lengert, Dmitry A. Gorin, Bogdan V. Parakhonskiy, Andre G. Skirtach

**Affiliations:** 1 Department of Molecular Biotechnology, Faculty of Bioscience Engineering, Ghent University, Coupure Links 653, 9000 Ghent, Belgium; lengertkatrin@mail.ru (E.V.L.); bogdan.parakhonskiy@ugent.be (B.V.P.); andre.skirtach@ugent.be (A.G.S.); 2 Educational Research Institute of Nanostructures and Biosystems, Saratov State University, Astrakhanskaya 83, 410012 Saratov, Russia; gorinda@mail.ru

**Keywords:** layer-by-layer membranes, microcapsules, induced release, permeability of membranes

## Abstract

Membranes are important components in a number of systems, where separation and control of the flow of molecules is desirable. Controllable membranes represent an even more coveted and desirable entity and their development is considered to be the next step of development. Typically, membranes are considered on flat surfaces, but spherical capsules possess a perfect “infinite” or fully suspended membranes. Similarities and transitions between spherical and flat membranes are discussed, while applications of membranes are also emphasized.

## 1. Introduction

Many scientifically relevant and industrially applicable processes are carried out using membranes as an active component [1]. In a typical configuration, membranes are deposited on a flat substrate and are operated to separate the two subcompartments, for example below and above the membrane functionalized surface. And this approach certainly has merits—the molecules in both compartments are separated statically and they can intermix dynamically if the flow through the membranes is controlled. Understanding of physico-chemical processes of the flow trafficking of molecules through the membranes is important for understanding of the principles of operation of the membranes, while, on the other hand, a number of industrial processes heavily rely on membrane separating molecules between two or more compartments.

In addition, the most abandoned living organism—a cell—is also protected by a membrane. In this case a very special membrane, which actually allows some proteins and ions to pass through and some proteins and ions to be filtered out.

In this mini-review, we provide insights into polymeric (mostly layer-by-layer) and lipid membrane from the point of view of creating remotely controllable membranes. Assembling of polymeric layer-by-layer (LbL) membranes is done through a sequential deposition of positively and negatively charged polyelectrolytes based on the electrostatic interaction, which is weaker than the covalent bonding. Versatility of this technique stems from the fact that virtually any material can be adsorbed or incorporated into the layers. Cross-linking is a technique used in chemistry, biochemistry, and even in cell biology (for crosslinking lipids in the cell surface). Cross-linking of LbL membranes allows for enhancing their stability and control the rejection [2] and it can also attain desirable properties of rejection and permeability. The stability of layer-by-layer membranes is the most desirable property, especially when the feedwater enfeebles the ionic bonds due to having high ionic strength. In this case, the cross-linking becomes essential. There are a number of cross-linking techniques includes chemical cross-linking (promoted by cross-linking agents such as terephthalaldehyde and glutaraldehyde) [3], UV-stimulated cross-linking [4], and heat-induced cross-linking [5]. The final cross-linked LbL membranes demonstrate improved rejection, enhanced chemical resistance, mechanical strength, and long-term stability.

The LbL membranes can be described in terms of their applications. The common application areas include water treatment [6], diffuse evaporation [7], fuel cell [8], and separation [9]. In the water treatment process, layer-by-layer membranes exhibit increased water permeability and relatively sufficient salt rejection. It is due to capability of formation of LbL multilayers with ultrafine thickness, which increases the water permeability, and capability of dense multilayers to moderate membrane selectivity because it is highly charged and have the small effective pore size, in some cases less than 1 nm [6]. Also, polyelectrolyte LbL membranes demonstrate long-term stability, which can be tuned by choosing polyelectrolyte type and membrane support [10]. Moreover, LbL membranes usually collect charges that may enhance the rejection because of Donnan exclusion. Additionally, the LbL method can be easily combined with other techniques to produce a new functional membrane, due to the facilities of functional groups in polyelectrolyte molecules. For instance, the layer-by-layer membranes for forward osmosis (FO) can be functionalized with silver (Ag) nanoparticles in order to create a novel FO membrane with antibacterial properties [11]. In the case of diffuse evaporation, layer-by-layer membranes can yield high permeate flow due to its ultrathin layers, and the selective layer which is resistant to organic solvent. In a fuel cell, LbL membranes can promote more efficient process of fabrication due to proton transfer in comparison with traditional techniques (spraying, paining, ink-jet printing, and so on). Moreover, LbL membranes, combined with other materials, can enhance catalyst utilization rate as well as enhance the fuel cell performance. In the case of separation, due to combination of a high flux with a high selectivity, LbL membranes have potential applications in different separation areas such as nanofiltration, solvent resistant nanofiltration, reverse osmosis, gas separation, and forward osmosis [9].

The polyelectrolyte multilayered sealed microchambers developed by M. Kiryukhin et al. [12,13,14] represent one more particularly interesting aspect of LbL membranes. The using of stimuli-responsive polyelectrolyte layers combined with metal nanoparticles in its composition allows it to attain changeable permeability of microchambers in response to various external factors (pH, ionic environment, chemical and biological stimuli, temperature, light, magnetic field, and ultrasound). These controllable membranes in form of sealed microchambers are attractive candidate for use in solid-state delivery systems and micropackaging.

**Figure 1:**
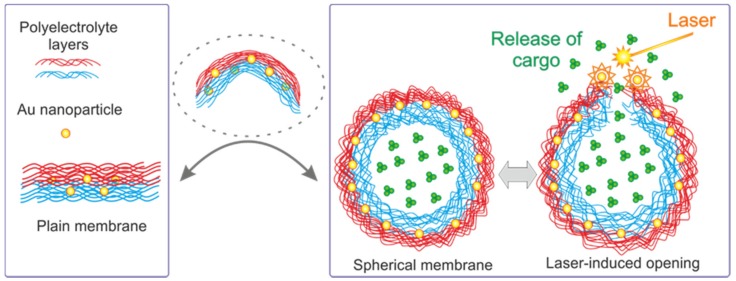
Schematic of a transition from flat membranes (real membranes depicted in the left-hand rectangle) to fully suspended membranes of microcapsules.

Spherical capsules are introduced as an “ideal” membrane, which is freely suspended in an aqueous solution and allows perfect understanding of how molecules constituting membranes interact with each other. Interestingly, a transition from spherical membranes of capsules to membranes adsorbed on flat surfaces and vice versa can be thought about (Figure 1). Thus, by studying and developing polymeric capsules one gets an instant insight into the function of molecules constituting the membrane. The same approach has been applied in the area of lipid membranes, where a transition from liposomes or giant liposomes to perfectly sealable flat membranes has been routinely applied.

Recent articles [15,16,17] provide detailed and comprehensive reviews about LbL capsules, microchambers, and films from the point of view of its remote controlling properties and various stimuli inducing its permeability changes and opening. In this review, we look at spherical and flat membranes from a unifying stand-point of view. In addition, we have also identified and described different stimuli, which can be used for controlling the permeability, encapsulation and release from polymeric capsules and liposomes, and their flat-surface analogues. Moreover, here we paid special attention to such significant LbL membrane components as metal and metal oxide nanoparticles, which endow membranes with a wide range of controllable properties include magnetic field-, ultrasound- and laser-responsive properties. The influence of nanoparticles type, concentration, distribution in membrane shell, character of aggregation on the light absorption, and membrane permeability were emphasized in this review.

## 2. Remote Control of the Permeability of Membranes—Release of Encapsulated Cargo from Capsules

There is a number of ways to stimulate control of the permeability of polyelectrolyte membranes, and it is possible to classify the types of external influence. The external stimuli (pH, ionic strength, solvent, and temperature) affect the permeability of the capsule shells in a reversible way by creating tiny pores in the polymeric structure and allowing or preventing the diffusion of molecules. These stimuli usually are reversible and can be useful for encapsulation under in vitro conditions, but present a high limitation for the release of the drugs in vivo.

Thus, in drug delivery and controllable release, external remote triggers are very relevant. Remote release techniques are irreversible since they lead to irreversible permeability changes. Such methods are intensively used for drug delivery, because they do not alter the chemical composition of the environment and therefore could be conducted under physiological conditions, where changing pH or ionic strength are not a viable option.

Further we will focus on stimuli, which affect the permeability of the capsules membrane in an irreversible way. Light irradiation treatment is a widely spread technique in medicine. There is a number of medical devices, used in photodynamic therapy, laser surgery, tissue modification, etc., giving a chance to apply optical control for drug release purposes. For this reason, the using of the optical radiation is promising for remote control of the microcapsules shells permeability. In order to enhance the capsule shell sensitivity to optical radiation, certain wavelengths are used, matching the strongest absorption of dyes or surface plasmon resonance of nanoparticle. An especially interesting application of such approach is remote initiation and manipulation of intracellular activity [18,19,20,21]. Therefore, this new generation of triggered nanocapsules opens a broad area for various potential applications, where a selective release is requested.

The effect of release by different polyelectrolytes types of the functional materials is caused by the nanoparticles such as TiO_2_, Ag, Au, Fe_3_O_4_ dyes, or polymers, as well as the number of layers affects the membrane permeability [22]. All data about the membrane composition, type of the modification, way of the influence is summarized in the Table 1.

## 3. Release Stimulated by Laser and IR Light

The light influence effect is based on the light adsorption by nanoparticles or polymers with the following transformation of this energy to the heating of surrounding environment, thus causing structural changes of the microcapsule shell. Detailed review by Parakhonskiy et al. [47] described the theory and practical application of this effect.

The effectivity of this method depended on the type of capsule morphology, type of nanoparticles, their distribution, and the laser wavelength changing the polymer conformation. More widely spreading is light energy being converted into heat [48,49] by nanoparticles [27]. The amount of heat generated can be carefully controlled by tuning laser intensity and illumination time. For example, when irradiated by a continuous wave laser, the nanoparticles will act as local heaters, causing a heat gradient in the surrounding tissue. Here it is important to distinguish between a “global” versus “local” temperature rise. As it was mentioned above, it is undesirable to raise global temperature of biological matter. However, under appropriate illumination conditions, heating can be locally induced without affecting the cell or tissue. It is this localized temperature rise that has been used for inducing transient [50] permeability of the polymeric shell of capsules, thus achieving controlled release of the pharmaceutical cargo. Increasing of the concentration of nanoparticles or augmenting the laser power density can lead to increased heat accumulation and therefore to explosive release or activation of microcapsules.

## 4. Laser Induced

**Figure 2:**
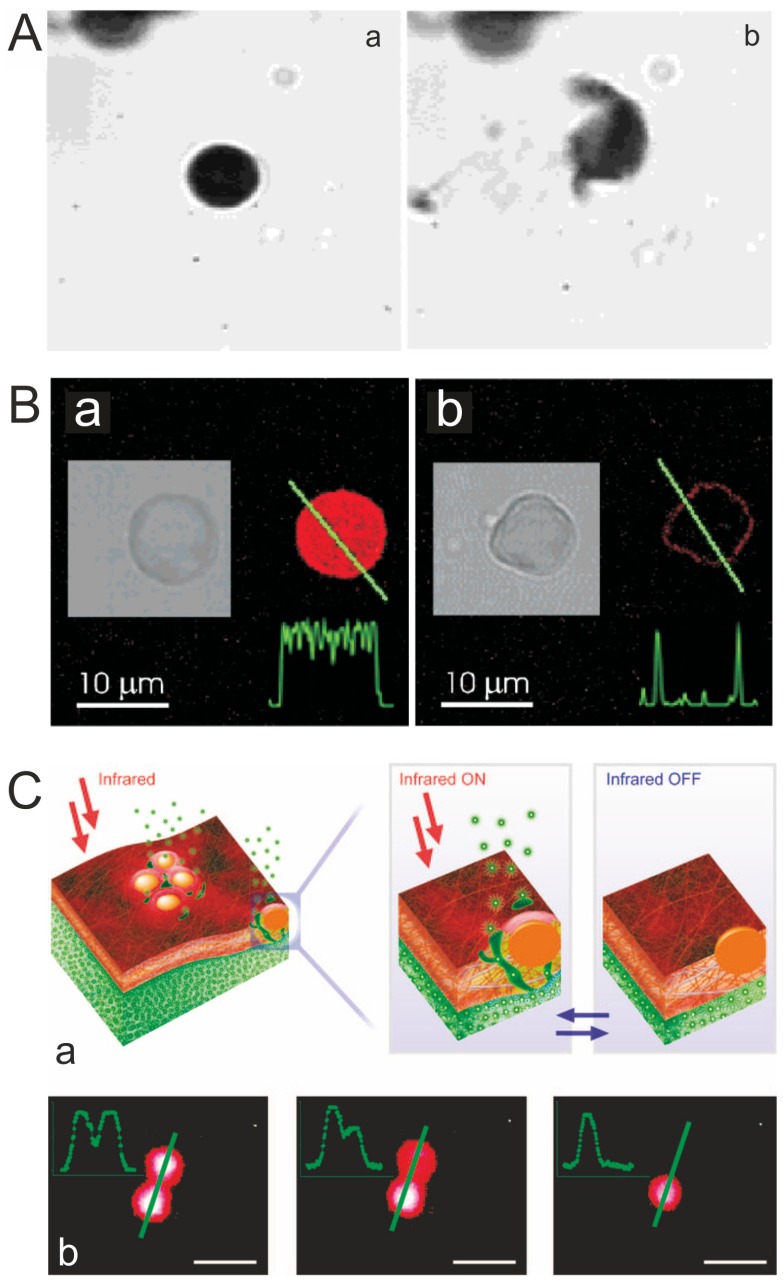
(**A**) The Ag-nanoparticle-doped capsule is intact before the interaction (a), and after the interaction (b) with the laser beam of 25 mW [23]. Copyright 2004 American Chemical Society; (**B**) Confocal microscope images demonstrating remote release of encapsulated cargo from a polyelectrolyte multilayer capsule containing metal nanoparticles in its walls [48]. Copyright 2005 American Chemical Society. (**C**) Remote release from microcapsules triggered by laser-induced opening channels on microcapsule membrane modified with nanoparticles. Profiles in the left upper corner are drawn along the green line. Scale bars correspond to 5 μm [50]. Copyright 2008 American Chemical Society.

The laser induced release can be non-invasive (by appropriate choice of wavelength), and offers high temporal and spatial control. Articles [23,48,51,52,53] studied the destruction of capsules by IR laser (wavelength 830 nm), containing 806 IR dye and silver nanoparticles obtained by the silver mirror reaction. Capsules of PAH/PSS were not destroyed by the laser radiation, since the PAH and PSS do not have absorption bands near the infrared region of the spectrum. Adding metal particles or molecules of the dye in the capsule shell results in the absorption of radiant energy (Figure 2), which lead to completely destruction of capsules (Figure 2A), its deformation (Figure 2B) [48]**,** or changing permeability of the shell without changing the capsules shape (Figure 2C) [50]. Figure 2B remote release of encapsulated rhodamine-labeled PSS polymers from a polyelectrolyte multilayer capsule containing gold sulfide core/gold shell nanoparticles in its walls. Fluorescence intensity profiles along the line through the capsule show that it is filled with fluorescent polymers before (a) and empty after (b) laser illumination. After the release of encapsulated polymers, the leftover fluorescent intensity is observed only in the walls of the capsule, (b). Insets show black and white transmission microscope images of the same capsule. Incident intensity of laser diode operating at 830 nm was set at 50 mW [48]. Figure 2C demonstrates a remote release from microcapsules: (a) schematics of nanoparticle functionalized polymeric nanomembranes opening channels upon laser illumination; (b) a polymeric microcapsule shell acts as a reversible nanomembrane. Upon laser light illumination the microcapsule (left image) partially releases encapsulated polymers and reseals (middle). After the second illumination the microcapsule completely releases its content (right) [50].

These effects are possible to reach because the concentration, distribution, and type of the nanoparticles have a strong influence to adsorption properties of the capsules shell. Tuning these parameters to the laser wave length results in an increase or decrease of affectivity of the irradiation influence (Figure 2B,C).

**Figure 3:**
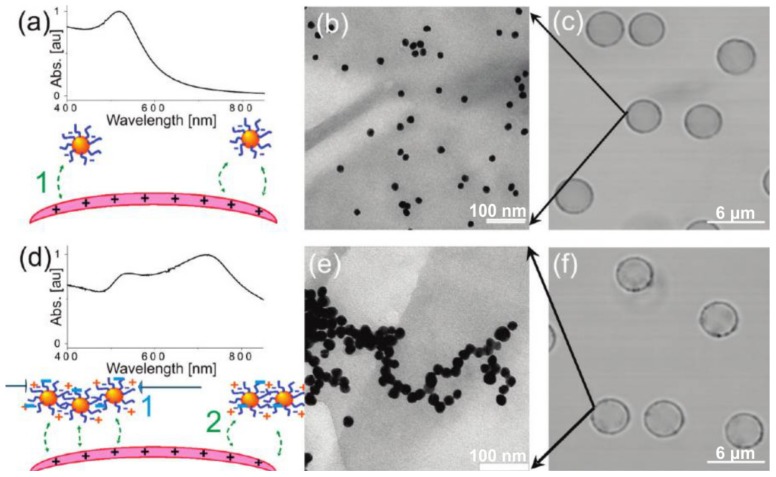
The influence of NPs distribution on absorption spectrum of microcapsules with NPs-modified shell. (**a**) Absorption spectrum for nonaggregated NPs and corresponding, (**b**) TEM image of nonaggregated NPs on a microcapsule, and (**c**) CLSM image of microcapsules; (**d**) Absorption spectrum for aggregated NPs and corresponding, (**e**) TEM image of aggregated NPs on a microcapsule, and (**f**) CLSM image of microcapsules [54]. Copyright 2010 American Chemical Society.

Nanoparticle adsorption at low [54] or high [18,55] concentrations is a key aspect in controlling not only heat generation around microcapsules (and thus release [51,56]), but also the permeability of microcapsules [56,57]. It is possible, for example, to control aggregation of particles by the premixture with polyelectrolyte with opposite charge to the particles. The difference in concentration and aggregation give difference in adsorption spectra of the capsules shell and as result have a strong influence to the laser irradiation sensitivity. Examples demonstrated on the Figure 3 [54]. This figure shows the distribution control of presynthesized gold nanoparticles (NPs) by adsorption onto polyelectrolyte multilayer capsules in the low concentration limit. The absorption spectra of nonaggregated NPs and aggregated NPs in microcapsule shell are compared [54].

Another way to control the nanoparticles aggregation is using template matrixes with predefined structure like porous particles. The type of template used has a strong influence on morphology and structure of resulted capsule and character of formation and distribution of nanoparticles in capsule shell [58]. The effect of laser radiation on the capsule, containing Ag and Au nanoparticles, was investigated [51,52]. Also in this work, capsules based on calcium carbonate microparticles and based on polystyrene cores were compared. The layer of silver nanoparticles was prepared by reacting a silver mirror during the formation of capsules and the adsorption of a mixture of separately synthesized nanoparticles and solution of an anionic polyelectrolyte. Attempts to break the infrared laser with a wavelength of 830 nm capsule layer, nanoparticles, which were formed by silver mirror reaction, did not give results. According to the authors, this is due to two facts. Maximum absorption of nanoparticles in the range of wavelengths 380–500 nm. Moreover, silver synthesized in the shell layer forms sufficiently dense layers. Destruction of capsules can be achieved only by using solid-state laser with a wavelength of 532 nm and a power of 100 mW. At the same time, the capsule shells containing nanoparticles, which adsorb sol, radiation, could be destroyed by the semiconductor infrared laser with a wavelength of 830 nm as the silver nanoparticles have sufficient absorption at this wavelength. Furthermore, it was found that capsules based on the calcium carbonate template, destroyed at lower output power in comparison with the capsules formed on polystyrene microparticles. This is due to the nature of the transfer of silver nanoparticles on the core: on polystyrene particles nanoparticles form a uniform layer, while in the case of calcium carbonate nanoparticles adsorb in pores and form clusters. It was shown, that the minimum power required to break capsules depends not only on the nature of the core, but the mass of adsorbed metal. Thus, with increasing mass of gold, the reduced laser power is enough for destruction of capsule, for the same capsules produced on different cores. The sensitivity of the laser in capsules synthesized on polystyrene cores slightly higher than sensitivity in capsules prepared on CaCO_3_ cores.

Another way to functionalize the capsule shell is using a light-sensitive polymer in the shell composition. Bedard and co-workers reported on the light-induced shrinking and encapsulation of a fluorescently labeled polymer in an azobenzene modified microcapsule that is highly resistant to thermal changes [53]. The efficient encapsulation of a labeled polymer in capsules was shown to occur when a mixture of dye and hollow shells was exposed to an intense light source. This was attributed to changes in the permeability of the shell following exposure to light. It was shown that the effectiveness of this encapsulation method, based solely on an optical approach, increases with the duration of irradiation time, as well as for larger fluorescent molecules. This approach is promising for the development of new optically active systems with applications in material science and electronics.

Although polymeric membranes represent a perfect approach for separating and controlling the traffic of large molecules, small molecules and ions are typically controlled by a lipid membrane. And this is not surprising, because even cells regulate their action through lipid membranes and built-in channel proteins. Therefore, it is even more important to control the flow through such membranes.

**Figure 4:**
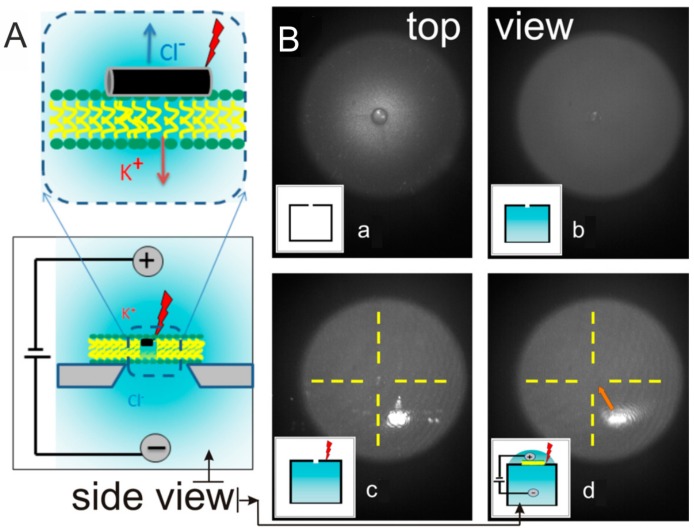
Scheme of the setup to perform the nanopore approach experiment for observation of traffic through lipid membranes. (**A**) Side view of the scheme of the experiment allowing observation and (**B**) images (top view) demonstrating the laser alignment on the glass chip [59]. Copyright 2014 American Chemical Society.

A setup, which allows one to observe traffic through lipid membranes is based on a so-called nanopore approach (Figure 4). Figure 4 demonstrates: (A) side view of the scheme of the experiment and (B) images (top view) that are demonstrating the laser alignment on the so-called chip (the glass surface on a cup that can be screwed on the nanopore device) with the following steps: (a) chip in air (the inset shows the side view schematics); (b) chip with water filling the lower chamber (the inset shows water in blue filling the lower chamber); (c) chip with water filling the lower chamber with laser focused at the top; (d) chip with water filling both chambers, the location of the opening is marked by the dashed yellow cross (the inset shows water filling both chambers and indicates presence of the laser is illuminating the top). In this approach, a small hole is drilled into the surface of the chip. Then a giant liposome is prepared and is left to be sucked by creating a pressure difference between the two compartments. Once a liposome is sucked into the hole on the surface of a chip, it “opens up” covering the hole completely. This blocks the ionic current and nothing can pass through the nanopore. Continuing the line of controlled membranes, it would be desirable to control the traffic of molecules or even more important ions through the nanopore. This has been done by shining a laser on the lipid membrane functionalized with gold nanoparticles and nanorods [59]. Theoretical modeling of molecular dynamics and transport in nanopores is described in [60]. Moreover, phase transition properties of lipid membranes can be controlled by plasmonic heating of gold nanoparticles [27,61,62,63].

Liposomes represents spherical-formed lipid membranes which were approved and successfully used for drug delivery applications [64]. The permeability of the lipid shell of a liposome can also be controlled by different external triggers include temperature, magnetic field, ultrasound, etc. in order to perform a controlled of drug release. The thermosensitive liposomes under mild hyperthermia (41–43 °C) can rapidly change their structure and form openings in their shell which allow the release of their payload. Due to these properties, thermosensitive liposomes can be applied for cancer treatment with drug release triggered by local heating induced by external stimuli. Sensitivity of thermo-responsive liposomes to type of stimuli depends on composition of liposome shell. Generally, release from liposomes can be activated by minimal invasive application of hyperthermia treatments include radiofrequency, microwave and high-intensity-focused ultrasound [65,66]. The heating effect can be enhanced by modifying liposomes with plasmon resonant nanoparticles [67,68,69]. The incorporation of iron oxide nanoparticles into liposome shells allows the use of alternative magnetic field for liposome opening, while heating of surrounding tissues are minimalized. These benefits are due to local heating of iron oxide nanoparticles followed opening of liposomes shells without environmental heating, which is important for treatment thermosensitive tissues [70,71].

## 5. US-Induced

Ultrasound is a method widely used for the synthesis of various nanomaterials, such as coating carbon nanotubes and noble metals and in various biomedical applications: destruction and fragmentation of contrast agents, gas release, polymer destruction, and in drug delivery [37]. Ultrasound offers an easy and fast way of inducing release from multilayered capsules and may be of interest to the biomedical field, for example, in topical application of ultrasound after subcutaneous injection of capsules. Ultrasound was used to destroy polyelectrolyte multilayer capsules as well as hydrogel capsules [36] based on PS (polystyrene) and CaCO_3_ cores. In this sense, nanoparticles were used to increase the density of microcapsule shells and ultrasound served as a trigger to release encapsulated material. Powers in the 100–500 W range at frequencies of 20 kHz were applied for capsule destruction. It was found that nanoparticles adsorbed on microcapsules affect the action of ultrasound on their shells. In was shown that US irradiation has a dramatic effect on the integrity of multilayered capsules, and leads to their destruction and the release of the encapsulated species.

In [72]**,** the effect of varying the intensity of the ultrasound and the duration of the impact on the integrity and membrane permeability of polyelectrolyte microcapsules contained the magnetite particles and ZnO_2_ particles [33] was studied. Ultrasonic effect dependent on power and time can lead to total destruction shells of the microcapsules. The presence of inorganic nanoparticles in the polyelectrolyte shell of the microcapsules increases their sensitivity to ultrasound exposure and simultaneously allows for concentration of the microcapsules by a magnetic field. Thus, ultrasound irradiation of microcapsules containing and not containing nanoparticles gives different results for the same exposure conditions. Ultrasound can trigger a chemical reaction catalyzed by nanoparticles embedded in the capsule shell. In [73]**,** authors described ultrasound-triggered disruption of the liposomes attached to the polyelectrolyte shell which can release the encapsulation material and accelerate the enzyme-catalyzed reaction inside the microcapsules and porous microparticles. The disruption of liposomes attached to micro particles was achieved by ultrasound under conditions similar to those used in medical ultrasound treatment; the key parameter was the density gradient around the lipid membrane. Ultrasound can be not only used for release, but also for encapsulation. The possibility of protein release from polymeric microcapsules by means of low-power high-frequency ultrasound was studied in [34]. The release efficiency is improved by the presence of gold nanoparticles in the microcapsule shell.

## 6. Microwave

Multilayered hollow capsules can also be functionalized with magnetic nanoparticles in order to enhance their therapeutic performance or to impart recognition by functional molecules to perform targeted delivery. Therefore, magnetic fields find increasing application in bio-medicine and drug delivery. Magnetic particles can be produced by calcination of the core–shell magnetite particles at elevated temperatures. Not all materials are compatible with magnetic particles: the solubility of magnetic particles at pH 1 makes melamine formaldehyde (MF) unsuitable, thus leaving, for example, PS, silica, or carbonates as a material of choice. Besides utilizing magnetic particles for targeting techniques, they can as well be used to affect the permeability of microcapsules by applying external oscillating magnetic fields and release encapsulated materials upon request. Ferromagnetic cobalt nanoparticles containing a layer of gold (Co/Au) were incorporated into the assembly of PSS and PAH polyelectrolyte multilayer shells of microcapsules. Subsequently, application of alternating magnetic fields with frequencies of 100–300 Hz and 1200 Oersted strength resulted in increased permeability and, thus, release of the encapsulated content. Lu and coworkers showed that magnetic field affects the permeability of microcapsules by acting on aggregates of nanoparticles [40]. The permeability of the (PSS/PAH)4(PSS/CoAu)1(PSS/PAH)6 capsule for FITC-dextran before applying alternating magnetic fields is negligible; FITC-dextran is blocked from diffusion into the capsules. After applying an alternating electromagnetic field to the capsule/FITC-dextran mixture for 30 min, the capsules became permeable. Although the magnetic activation of microcontainers is a good candidate for controlled drug delivery, the long exposure time and the strong magnetic field required to permeabilize the capsules lead to an increase in the temperature which is problematic for loading sensitive or instable proteins or other molecules into the capsules.

## 7. Mechanical

Caruso et al. developed capsules that release carbon nanotubes upon mechanical rupture [74]. This technology could be extended to a variety of applications including safer and longer-lifetime batteries. Very recently, a new method consisting of release upon mechanical deformation of polyelectrolyte multilayer capsules has been developed by combining fluorescence microscopy with atomic-force microscopy (AFM) [75]. Using the colloidal force probe technique, we studied mechanical release from polyelectrolyte multilayer capsules filled with fluorescently labeled dextran molecules upon mechanical deformations. This experimental approach allows independent measurement of release and plastic deformation thresholds. It was shown that release of fluorescent content is triggered above 18% of relative capsule deformation. The capsule plastic deformation was found to occur also above 18% total capsule deformation. The quantification of release upon mechanical deformation provided information that could help to design microcapsules with optimal mechanical properties for intracellular delivery in particular and delivery in general. Using colloidal probe AFM combined with quantitative fluorescence microscopy; [76] investigated mechanical properties and release from CaCO_3_-templated polymeric capsules made of biodegradable polymers. The mechanical and release properties of these capsules were studied in comparison with those of CaCO_3_-templated capsules composed of synthetic polymers. Furthermore, we assessed the influence of the number of polyelectrolyte LbL layers on the mechanical properties and release from biodegradable capsules.

## 8. Conclusions

Membranes have captivated the attention of scientists for a long time due to their usefulness and broad range of applications. Their applications include coatings, membranes, capsules, and other delivery vehicles. A growing area of application is that in membranes, which possess different functionalities, like purification, separation, etc. For these interesting practical applications, there is a limited understanding of physico-chemical properties of the membranes, partially due to the fact that polymers are adsorbed and are in their so-called “glassy” state once adsorbed on substrates. Indeed, most of the membranes are flat or adsorbed on flat surfaces, and they are connected to these substrates. On the other hand, some of the most interesting aspects of membranes can be studied if these are suspended in an aqueous solution. Microcapsules represent a perfect example of a system completely suspended in a liquid, and therefore they have provided a fertile ground for understanding and controlling their permeability and other physico-chemical characteristics. As a result, interesting phenomena have been developed allowing understanding of the principles of operation and leading to new industrially relevant applications.

## Figures and Tables

**Table 1:** Summary of different mechanisms used for controlling the permeability between different types of membranes. Further, abbreviations of polymers used as membrane materials: PAH, poly(allylamine hydrochloride); PSS, poly(4-styrenesulfonic acid) sodium salt); MF, melamineformaldehyde; HA, hyaluronic acid; PLL, poly(l-lysine); DES, dextran sulfate; PVS, poly(vinyl sulfate); PLGA, poly(lactic-co-lycolic acid); PDADMA, poly(diallyldimethylammonium chloride); P(Am-DDA), poly(acrylamide-co-diallyl-dimethylammonium chloride.

Technique of Release	Membrane Material	Functionalization	Core/Template
Laser-induced, laser heating	PAH/PSS	Ag nanoparticles [23], Au nanoparticles [24], Bacteriorhodopsin [20]	MF latex particles [23,24], CaCO_3_ particles [20]
	Alginate	Ag nanoparticles [25]	CaCO_3_ particles [25]
	Phospholipids [26]	Au nanoparticles [27]	–
	Poly(arginine)/DES	Fe_3_O_4_ nanoparticles and Au nanoparticles [28]	CaCO_3_ particles [28]
NIR irradiation	PAH/PSS Witepsol W 31 (W/O/W emulsion) [29], (HA/PLL)24/PLL [30]	Gold nanoparticles [30,31,32]	Porous polycarbonate membrane [31], CaCO_3_ particles [32]
Ultrasonic irradiation	PAH/PSS	ZnO particles [33], Au nanoparticles [34], Fluorescent carbon dots (CDs) [35]	CaCO_3_ particles [33,34,35]
	Silver alginate	Ag nanopartiles [36]	CaCO_3_ particles [36]
pH-induced	PAH/PSS	–	CaCO_3_ particles [37,38], PLGA [39]
	PLL/DES [39]	–	PLGA [39]
	PAH/PVS [38]	–	CaCO_3_ particles [38]
	PAH/DES [38]	–	CaCO_3_ particles [38]
	PAH/nucleic acid [40,41]	–	CaCO_3_ particles [40,41]
	Alginate/Poly-L-ysine	Au nanoparticles [42]	Fe-SiO_2_ [42]
Magnetic Field	PDADMA/PSS [43], PSS/PAH/P(Am-DDA) [44]	Iron oxide nanocubes [44]	Fe(CO)_5_@SiO_2_ [43], CaCO_3_ particles [44]
Microwave irradiation	PAH/PSS	Au nanoparticles [45]	CaCO_3_ particles [45]
Induced by nonionic surfactant	Chitosan/alginate [46]	–	Liposomes [46]

## Abbreviations

LbL membrane	layer-by-layer membrane
FO membrane	forward osmosis membrane
W/O/W emulsion	water-in-oil-in-water emulsion
UV	ultraviolet
NIR	near infrared
CLSM	confocal laser scanning microscopy
TEM	transmission electron microscopy
AFM	atomic-force microscopy
PS core	polystyrene core
NP	nanoparticle
CD	carbon dot
FITC	fluorescein isothiocyanate
MF	Melamine formaldehyde
HA/PLL	Hyaluronic Acid/Poly(l-lysine)
DES, DS	dextran sulfate
PDADMA	poly(diallyldimethylammonium chloride)
P(Am-DDA)	poly(acrylamide-co-diallyl-dimethylammonium chloride)
PAH	poly(allylamine hydrochloride)
PSS	poly(4-styrenesulfonic acid) sodium salt
